# Head and Neck Cancer Patient Population, Management, and Oncologic Outcomes from the COVID-19 Pandemic

**DOI:** 10.3390/curroncol31010029

**Published:** 2024-01-11

**Authors:** Julie R. Bloom, Carlos Rodriguez-Russo, Kristin Hsieh, Daniel R. Dickstein, Ren-Dih Sheu, Mayuri Jain, Erin Moshier, Jerry Liu, Vishal Gupta, Diana N. Kirke, Scott Roof, Krzysztof Misiukiewicz, Marshall Posner, Richard Bakst, Kunal K. Sindhu, Sonam Sharma

**Affiliations:** 1 Department of Radiation Oncology, Icahn School of Medicine at Mount Sinai, New York, NY 10029, USAkunal.sindhu@mountsinai.org (K.K.S.); 2 Department of Population Health Science and Policy, Tisch Cancer Institute Biostatistics Shared Resource Facility, Icahn School of Medicine at Mount Sinai, New York, NY 10029, USA; 3 Department of Otolaryngology-Head and Neck Surgery, Icahn School of Medicine at Mount Sinai, New York, NY 10029, USA; 4 Department of Hematology/Oncology, Icahn School of Medicine at Mount Sinai, New York, NY 10029, USA; 5 Icahn School of Medicine at Mount Sinai, Tisch Cancer Institute, New York, NY 10029, USA; 6 Department of Radiation Oncology, Summit Health, Berkeley Heights, NJ 07922, USA

**Keywords:** head and neck cancer, radiation therapy, COVID-19, induction chemotherapy, gender disparity

## Abstract

The COVID-19 pandemic precipitated drastic changes in cancer care. Its impact on the U.S. head and neck cancer population has yet to be fully understood. This study aims to understand the impact of pandemic-related changes on the head and neck cancer population. An observational study of head and neck cancer patients at a single institution during the spring of 2020 and 2019 was performed. Clinical characteristics and survival outcomes were analyzed. In 2020, 54 head and neck cancer patients were evaluated in the department of radiation oncology vs. 74 patients seen in 2019; 42% of the patients were female in 2019 versus 24% in 2020 (*p* = 0.036). The median follow-up time was 19.4 and 31 months for 2020 and 2019, respectively. After adjusting for stage, the relapse-free survival probability at 6 and 12 months was 79% and 69% in 2020 vs. 96% and 89% in 2019, respectively (*p* = 0.036). There was no significant difference in the overall survival, with 94% and 89% in 2020 and 2019, respectively (*p* = 0.61). Twenty-one percent of patients received induction chemotherapy in 2020 versus 5% in 2019 (*p* = 0.011); significantly more treatment incompletions occurred in 2020, 9% vs. 0% in 2019 (*p* = 0.012). Moreover, the stage-adjusted RFS differed between cohorts, suggesting head and neck cancer patients seen during the initial wave of COVID-19 may experience worse oncologic outcomes.

## 1. Introduction

On 11 March 2020, the World Health Organization declared COVID-19 a global pandemic. In the wake of this declaration, non-emergent outpatient care, including routine oncologic screening, diagnostic workup, and standard of care management, was altered. National consensus recommendations from the Centers for Medicare and Medicaid Services and the American College of Surgeons advised the cancellation of non-emergent surgical procedures, which in some cases included “low-risk” cancer surgeries [1,2]. With the perceived high risk of COVID-19 transmission during surgical procedures, patient evaluation with surgical processes (including flexible sinonasal endoscopy) and diagnostic imaging affected oncologic surveillance [3,4]. As a result, several international consortium groups identified an urgent need for new practice recommendations regarding head and neck oncology care [5,6]. Examples of recommended alterations in care included delayed surgical therapy for early stage malignancy, the avoidance of free flap reconstruction, and in the case of radiation therapy, increased use of hypofractionated radiation therapy [5,6,7].

While the impact of COVID-19 on treatment patterns for certain malignancies has been documented, its clinical impact upon the care of patients in radiation oncology has not yet been comprehensively assessed. For example, the impact on patients seen in surgical oncology specialties has been analyzed with results demonstrating that 40% of patients experienced surgical delays due to the COVID-19 pandemic in New York City, where the pandemic greatly affected cancer centers early in 2020 [8]. Additionally, of the patients recommended to undergo adjuvant therapy, 33% did not complete their adjuvant therapy [8]. Ultimately, the impact on patients with head and neck cancers seen and treated within departments of radiation oncology has not been explored. Given the known relation between a delay in cancer care and worse clinical outcomes [9,10,11], the authors hypothesized that the potential delay in diagnosis and treatment due to the COVID-19 pandemic may lead to worse oncologic outcomes [3,12]. 

The goal of this study was to understand the clinical impact of the COVID-19 pandemic on treatment patterns and clinical outcomes of patients with head and neck cancer. We hypothesized that treatment patterns during the height of the pandemic, March through June of 2020, versus (vs.) the same time period the year prior would differ and potentially precipitate worse clinical outcomes in head and neck cancer patients.

## 2. Materials and Methods

### 2.1. Study Design and Population

In this retrospective review, radiation treatment parameters for head and neck cancer patients treated within the department of radiation oncology at a single large academic institution in New York City were compared for the initial peak wave of the pandemic to the same time frame 1 year prior (1 March to 30 June of 2020 vs. 1 March to 30 June of 2019). Head and neck disease sites were obtained and categorized by ICD10 code and included hypopharynx, larynx, nasopharynx, oral cavity, oropharynx, orbit/skull base, salivary, sinonasal/paranasal, thyroid, and other. Approval of this study was obtained by the Institutional Review Board at our institution.

### 2.2. End Points and Statistical Analysis

Descriptive analysis of the patients’ baseline demographic and clinical characteristics was performed, stratified by consultation year. Categorical variables at baseline were summarized as the frequency (n) and percentage (%) and compared between consultation years using the chi-squared test or Fisher’s exact test where appropriate. Continuous variables at baseline were summarized as the mean (standard deviation) and median (interquartile range) and compared between consultation years using a *t*-test or Wilcoxon rank-sum test, respectively.

The method of Kaplan–Meier was used to estimate the distributions of time to event outcomes stratified by the year of consultation, and their distributions were compared using the log-rank test. The overall survival was defined as the time from treatment initiation to death by any cause or to the last follow-up date. The cohort for overall survival analysis included patients whose treatment initiation was not missing and whose cancer stage was not defined as benign. Relapse-free survival (RFS) was defined as the time from treatment initiation to the relapse date, death by any cause (if the relapse date was missing), or to the last imaging date. The cohort for RFS included patients whose treatment initiation date was not missing or last imaging date was not missing. RFS was adjusted to account for stage and sex using the inverse probability weights method [13,14]. Hypothesis testing was two-sided and conducted at the 5% level of significance. All statistical analyses were conducted using SAS v9.4. (SAS Institute Inc., Cary, NC, USA).

## 3. Results

### 3.1. Patient Population

From 1 March to 30 June 2020, 54 head and neck cancer patients were seen in consultation within the department of radiation oncology, a 27% reduction from 2019 during which 74 patients were seen in consultation (Table 1). The median age for the 2019 cohort and 2020 cohort was 61.5 and 62.5, respectively. In 2019, 42% of patients seen in consultation were female vs. 24% of patients in 2020 (*p* = 0.036). Of the patients seen for a consultation, 97% of patients in 2019 and 96% of patients in 2020 received radiation therapy. The area deprivation index—a ranking system of neighborhoods by socioeconomic disadvantage accounting for factors of theoretical domains such as income, education, employment, and housing quality—was not significantly different between 2019 and 2020 [15].

Of the patients treated in 2020, 59% were treated with definitive radiation therapy with or without chemotherapy vs. 54% in 2019 (*p* = 0.71). Additionally, 4% of patients in 2020 received palliative radiation therapy vs. 3% in 2019 (*p* = 0.71). In 2020, 21% of patients received induction chemotherapy vs. 5% in 2019 (*p* = 0.011). The radiation dose received and fractionation regimen did not significantly differ between cohorts. Zero patients had treatments altered in 2019 vs. 9% of patients in 2020 (*p* = 0.012). Treatment alteration was defined as an unplanned break in radiation therapy or incompletion of their planned radiation therapy course defined at the start of radiation therapy. Of the patients who had treatment alterations in 2020, four patients did not complete treatment (*p* = 0.029). The number of patients enrolled in clinical trials did not differ significantly between cohorts. Two patients were diagnosed with COVID-19 while on treatment with no treatment interruption.

### 3.2. Survival Analyses

**Figure 1:**
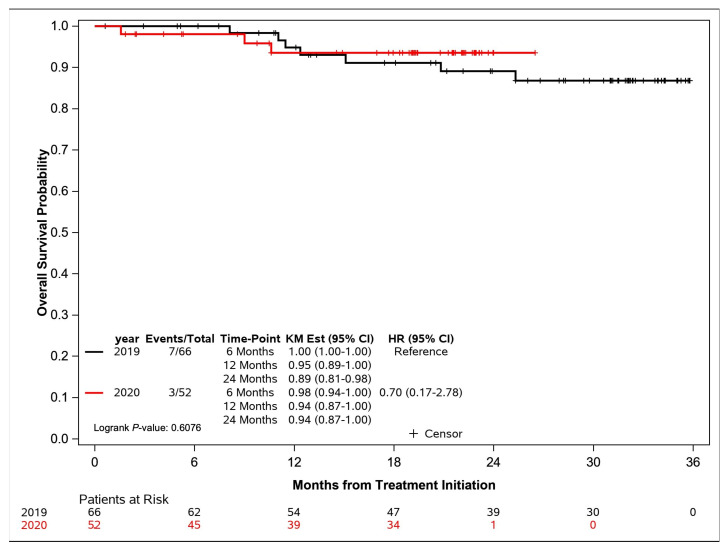
Overall survival for head and neck patients.

The median time to follow-up was 31 months for 2019 and 19.4 months for 2020. There was no significant difference in the unadjusted RFS and overall survival (Table 2). Of the 66 patients who started their treatment in 2019, ultimately, 7 (11%) patients died vs. 3 (6%) patients of the 52 patients who started treatment in 2020 (Figure 1), although the latter cohort’s follow-up interval is shorter. In 2019, the estimated overall survival probability at 6, 12, and 24 months from initiating treatment was 100%, 95%, and 89%, respectively. In 2020, the estimated overall survival probability at 6, 12, and 24 months from initiating treatment was 98%, 94%, and 94%, respectively (*p* = 0.61).

**Figure 2:**
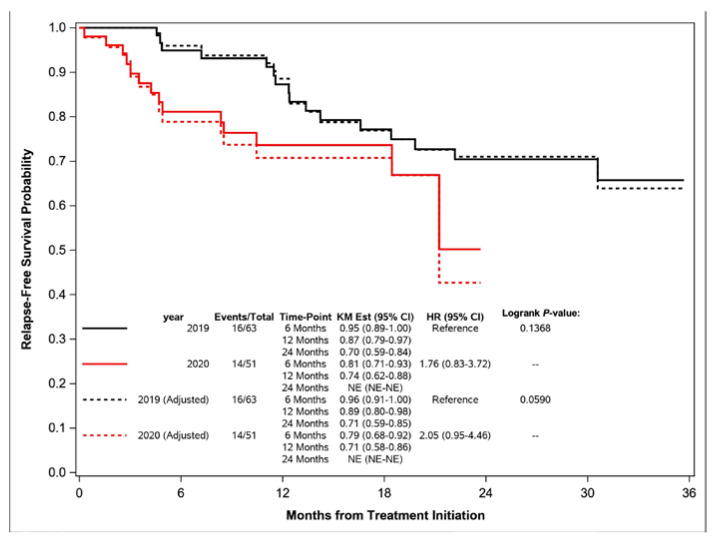
Relapse-free survival and stage-adjusted relapse-free survival for head and neck patients.

Of the 63 patients who started their treatment in 2019, 16 (25.4%) patients were diagnosed with a relapse during the follow-up period. Similarly, 14 (27%) of 51 patients were diagnosed with a relapse in the 2020 cohort. In 2019, the RFS probability at 6 and 12 months from initiating treatment was 95% and 87%, respectively (Figure 2). In 2020, the RFS probability at 6 and 12 months from initiating treatment was 81% and 74% (*p* = 0.14). After adjusting for stage, the RFS probability at 6 and 12 months for the 2019 cohort was 96% and 89%, respectively, and was 79% and 69% (*p* = 0.036) for the 2020 cohort, respectively. Additionally, after adjusting for stage and sex, the difference in RFS between cohorts remained significant (*p* = 0.05; Supplemental Figure S1).

### 3.3. Clinical Time Parameters

For the various time parameters—weeks from biopsy to consult, weeks from biopsy to treatment, weeks from surgery to treatment, weeks from consultation to simulation, weeks from simulation to treatment, and weeks from consultation to treatment—there were no statistically significant differences between the two cohorts (Table 3). The median weeks from biopsy to consultation was numerically higher in the 2019 cohort vs. the 2020 cohort, with the probability for consultation within 4 weeks of biopsy to be 0.51 in 2019 vs. 0.75 in 2020 (*p* = 0.0839). 

## 4. Discussion

In this single-institutional retrospective review evaluating treatment parameters and clinical outcomes for head and neck cancer patients during the spring of 2019 vs. spring of 2020 during the height of the COVID-19 pandemic, several differences between the cohorts were noted. While there was no delay noted in the measured time parameters, there was, ultimately, a significant difference in the stage-adjusted RFS. This difference did not translate into a difference in overall survival. Notable differences between the treatment patterns and population characteristics were observed including significantly more patients receiving induction chemotherapy in 2020 vs. 2019 and significantly more treatment incompletions in 2020 vs. 2019. Finally, there were disproportionately fewer females in the 2020 cohort in comparison to the 2019 cohort.

While there was no significant difference in the unadjusted overall survival or RFS distributions between the 2020 and 2019 cohorts, once adjusted for stage, the RFS probability at 6 and 12 months significantly differed (*p* = 0.036). One explanation for this difference could be due to the difficulty in accessing medical care during the COVID-19 pandemic and the delay in adequate therapeutic intervention prior to biopsy, this study’s initial time point reference [16]. The worse stage-adjusted RFS in 2020 is consistent with predictive models estimating excess morbidity among patients with cancer during the pandemic [12,17]. While there have been several studies exploring the influence of cancer as a comorbidity and how cancer may affect the severity of coronavirus, there has been little published to date on any difference in oncologic outcomes for cancer patients seen and treated during the initial wave of the COVID-19 pandemic [18,19]. To our knowledge, this is the first study to explore any differences in oncologic outcomes for patients treated during the initial wave of the COVID-19 pandemic compared to another similar cohort.

With respect to clinical treatments received, while there were no differences in the percentage of patients who received definitive vs. adjuvant RT, a significantly higher percentage of the 2020 cohort received induction chemotherapy, 21% vs. 5%, in 2019, (*p* = 0.011). In 2020, the patients who received induction chemotherapy had primaries including nasopharynx, larynx, oropharynx, hypopharynx, oral cavity, and sinonasal locations. The increased rate of induction chemotherapy and diverse primary profile of recipient patients may have been a strategy used to bridge patients whose surgeries were postponed or canceled while the patients waited to be scheduled for radiation therapy. This finding is concordant with other research demonstrating that in patients with ovarian and endometrial cancer, patients underwent neoadjuvant systemic therapy prior to debulking surgery due to the imposed restrictions on elective surgery as bridging to definitive surgical management [8]. With regards to radiation therapy treatment patterns, while there were no significant differences in dose or fractionation prescription, there were notably more treatment interruptions. Ultimately, 9% of patients in 2020 (*p* = 0.012) had treatment interruption, with 7% prematurely discontinuing treatment (*p* = 0.029) in 2020. Zero patients had treatment adjustment or discontinuation in 2019. Two patients’ deviations from the initially prescribed treatment were due to the concern for hospitalization and difficulties related to transportation in the setting of COVID-19. This finding is similar to published work in non-head and neck specialties, which suggest reasons for not completing treatment included medical limitations, decline of therapy due to COVID-19 related concerns, patients lost to follow-up, and social/financial reasons, among others [8]. Of two patients diagnosed with COVID-19 while on treatment, appropriate departmental adjustments were taken as precaution with no interruption or alteration in their treatment courses. Finally, systemic qualities such as time to event parameters and trial enrollment did not significantly differ between cohorts in contrast to some radiation oncology departments outside the United States and in other specialties [16,20,21,22,23,24,25,26,27].

Consistent with previously-reported disparities evident during the COVID-19 pandemic [28,29,30,31,32,33,34], the gender of the head and neck population significantly differed between cohorts with females comprising 42% of the population in 2019 and only 24% in 2020 (*p* = 0.036). This disparity is evident in other databases that demonstrate a decrease in the proportion of female patients presenting to medical centers during the pandemic vs. pre-pandemic [35,36,37]. It is possible the gender disparity demonstrated in this study is a downstream effect of the disproportionate burden placed on women during the pandemic including existing gender inequalities in the workforce, labor market segregation, and childcare responsibilities [32,33,34,38,39,40,41]. When considering patient characteristics’ including the area deprivation index, there was no difference in the area deprivation index between cohorts. This is in contrast to what the authors hypothesized given the experienced disparities during COVID-19 [40,42]. One explanation for this may be due to this academic institution’s location, easily accessible by public transportation within a 5-mile radius of neighborhoods with an area deprivation index of 10 (most disadvantaged groups). The similarity in the median area deprivation index may reflect the success of institutional efforts such as designating a radiation oncology-specific social worker and maintaining access to medical transportation services for disadvantaged individuals during this critical time. 

Finally, how can cancer centers adjust to potentially mitigate unforeseen external factors such as a global pandemic? Strategies to continue treatment with proven standard of care oncologic therapy should be maintained to the best of the medical center’s ability, including working to ensure definitive surgery and definitive chemoradiation therapy takes place for appropriate patients. Multidisciplinary conversations should be maintained to strategically optimize patients’ care plans when in resource limited settings. Lastly, utilization of supportive services can help facilitate patient compliance especially when unusual external factors are at play including an increased burden of childcare, transportation difficulties, and further economic constraints. 

The limitations of this study include the inherent limitations of a single-institution retrospective review with a small study population. These include a select patient population that may not be representative of the majority. Additionally, the various time parameters measured (such as time from biopsy to consult) may not explain causation. An important variable, which was not accounted for in this study, encompasses time from symptom onset to biopsy/treatment, as many patients delayed their initial presentation to a healthcare provider. This may be where the discrepancy in the two cohorts’ stage-adjusted RFS lies. Additionally, it would be interesting to note based on multidisciplinary discussion documentation, if there were patients initially planned for another type of oncologic therapy, such as surgery, who were delayed, and how this might impact their oncologic outcomes after they went on to receive radiation therapy; this is not explored in this research. Furthermore, the head and neck cancer patient population includes a heterogeneous cohort; so, although there was a difference in stage-adjusted RFS, further support in future studies with longer follow-up are needed to definitively conclude that patients seen during the pandemic exhibited worse clinical outcomes. 

## 5. Conclusions

In this single-institution retrospective review, the head and neck cancer patient population treated in March through June of 2019 vs. 2020 differed in notable ways. While the overall survival was similar between the two cohorts, the stage-adjusted RFS differed significantly. These findings suggest that head and neck cancer patients diagnosed and seen during the initial wave of COVID-19 experienced worse oncologic outcomes consistent with the initial predictive models. Additionally, treatment pattern differences such as those who received induction chemotherapy and treatment interruptions were observed in 2020. Finally, minority disparities including a decrease in female patients seen during the pandemic were noted. Larger, multi-institutional studies are needed to validate these findings. The authors fear that in regions where there were delays in oncologic care, including RT, the clinical consequences may be exaggerated. Future efforts should focus on preparing medical centers to ensure the delivery of essential cancer treatments and minimize non-traditional treatment strategies, particularly in the setting of curative treatments for head and neck cancer. Potential strategies to moderate these outcomes should be explored including shorter interval follow-up and higher suspicion for relapse should it be warranted. 

## Figures and Tables

**Table 1:** Characteristics of head and neck cancer patients.

Characteristic	2019 (N = 74)	2020 (N = 54)	*p* Value
Age (years)			
**Mean (SD)**	61.6 (13.6)	62.5 (14.6)	0.6916
**Median (Min, Q1, Q3, Max)**	61.5 (21, 56, 71, 87)	62.5 (26, 55, 75, 87)	0.5938
Gender, N (%)			
**Female**	31 (41.9)	13 (24.1)	0.0361 *
**Male**	43 (58.1)	41 (75.9)	
Stage, N (%)			
**Benign**	6 (8.1)	1 (1.9)	0.0726
**1**	15 (20.3)	9 (16.7)	
**2**	10 (13.5)	9 (16.7)	
**3**	12 (16.2)	19 (35.2)	
**4**	31 (41.9)	16 (29.6)	
Area Deprivation Index (ADI)			
**Mean (SD)**	3.4 (2.1)	3.4 (2.1)	0.8953
**Median (Min, Q1, Q3, Max)**	3 (1, 2, 5, 8)	3 (1, 2, 5, 9)	0.9536
Treatment Intent, N (%)			
**Adjuvant**	32 (43.2)	20 (37.0)	0.7113
**Definitive**	40 (54.1)	32 (59.3)	
**Palliative**	2 (2.7)	2 (3.7)	
Induction Chemotherapy, N (%)			
**No**	70 (94.6)	42 (79.3)	0.0114 *
**Yes**	4 (5.4)	11 (20.7)	
Trial, N (%)			
**No**	66 (89.2)	48 (88.9)	0.9571
**Yes**	8 (10.8)	6 (11.1)	
Dose Received (cGy)			
**Mean (SD)**	5995.9 (1551.7)	5953.8 (1512.0)	0.8804
**Median (Min, Q1, Q3, Max)**	6600 (1480, 5900, 7000, 10,000)	6600 (848, 5700, 7000, 7000)	0.9173
Fraction Received			
**Mean (SD)**	28.8 (8.5)	28.8 (8.2)	0.9860
**Median (Min, Q1, Q3, Max)**	30 (1, 28, 35, 35)	33 (4, 27.5, 35, 35)	0.9689
Treatment Receipt, N (%)			
**No**	2 (2.7)	2 (3.7)	1.0000
**Yes**	72 (97.3)	52 (96.3)	
Telemedicine visit, N (%)			
**No**	74 (100)	15 (27.8)	<0.0001 *
**Yes**	0 (0)	39 (72.2)	
Treatment discontinued, N (%)			
**No**	74 (100)	50 (92.6)	0.02964 *
**Yes**	0 (0)	4 (7.4)	
Total treatment duration (in days)			
**Mean (SD)**	42.8 (14.3)	41.4 (16.0)	0.6098
**Median (Min, Q1, Q3, Max)**	44 (5, 39, 49, 103)	44.5 (11, 36.5, 49, 120)	0.5244
Median follow-up (in months)			
**Median (95% CI)**	31.0 (27.9–32.1)	19.4 (19.0–22.1)	<0.0001 *
Treatment altered, N (%)			
**No**	74 (100)	49 (90.7)	0.0119 *
**Yes**	0 (0)	5 (9.3)	
Race, N (%)			
**African American/Black**	6 (8.1)	3 (5.6)	0.0560
**Arab**	0 (0)	1 (1.8)	
**Asian**	9 (12.2)	11 (20.4)	
**Caucasian/White**	33 (44.6)	22 (40.7)	
**Hispanic/Latino**	6 (8.1)	0 (0)	
**Not reported**	8 (10.8)	2 (3.7)	
**Other**	12 (16.2)	15 (27.8)	
Language, N (%)			
**English**	63 (85.1)	43 (79.6)	0.5626
**Spanish**	7 (9.5)	5 (9.3)	
**Other**	4 (5.4)	6 (11.1)	
Morphology, N (%)			
**Squamous cell carcinoma, NOS**	57 (77.0)	42 (79.2)	0.5956
**Adenoid cystic carcinoma**	2 (2.7)	3 (5.7)	
**Others**	15 (20.3)	8 (15.1)	

* *p* < 0.05.

**Table 2:** Overall survival and relapse-free survival clinical outcomes.

	2019 (N = 74)	2020 (N = 54)	Log-Rank *p* Value
Relapse-Free Survival (RFS)			
**Recurred, N (%)**	10 (15.9)	11 (21.6)	0.1368
**Local Relapse**	7 (11.1)	9 (17.6)	
**Distant Metastasis**	3 (4.8)	2 (3.9)	
**Deceased, N (%)**	6 (9.5)	3 (5.9)	
**Alive and Relapse-Free, N (%)**	47 (74.6)	37 (72.5)	
**Excluded, N (%)**	11 (14.9)	3 (5.6)	
**6-Month RFS [95% CI]**	0.95 (0.89–1.00)	0.81 (0.71–0.93)	
**12-Month RFS [95% CI]**	0.87 (0.79–0.97)	0.74 (0.62–0.88)	
**24-Month RFS [95% CI]**	0.70 (0.59–0.84)	Not Reached	
**Median [95% CI]**	Not Reached	Not Reached	
Overall Survival (OS)			
**Deceased, N (%)**	7 (10.6)	3 (5.8)	0.6076
**Alive, N (%)**	59 (89.4)	49 (94.2)	
**Excluded, N (%)**	8 (10.8)	2 (3.7)	
**6-Month OS [95% CI]**	1.00 (1.00–1.00)	0.98 (0.94–1.00)	
**12-Month OS [95% CI]**	0.95 (0.89–1.00)	0.94 (0.87–1.00)	
**24-Month OS [95% CI]**	0.89 (0.81–0.98)	0.94 (0.87–1.00)	
**Median [95% CI]**	Not Reached	Not Reached	

**Table 3:** Clinical time parameters.

Median Time Parameters [95% CI]	2019 (N = 74)	2020 (N = 54)	Log-Rank *p* Value
Weeks from Biopsy to Consult	4.0 (3.3–5.4)	3.4 (2.4–3.7)	0.0839
Weeks from Biopsy to Treatment	9.9 (8.0–12.0)	7.6 (6.7–10.0)	0.2984
Weeks from Surgery to Treatment	6.2 (6.0–7.6)	6.6 (5.7–7.9)	0.8263
Weeks from Consult to Simulation	1.8 (1.6–2.6)	1.8 (1.6–2.6)	0.9272
Weeks from Simulation to Treatment	1.7 (1.6–1.7)	1.6 (1.4–1.7)	0.2399
Weeks from Consult to Treatment	3.9 (3.4–5.0)	3.6 (3.0–5.1)	0.8787

## Data Availability

The data presented in this study are available on request from the corresponding author. The data are not publicly available due to institutional policies and protected patient data.

## Supplementary Materials

The following supporting information can be downloaded at: https://www.mdpi.com/article/10.3390/curroncol31010029/s1, Figure S1: Stage- and gender-adjusted relapse-free survival for head and neck cancer patients.
