# Supplementary figures and images for: Head and Neck Cancer Patient Population, Management, and Oncologic Outcomes from the COVID-19 Pandemic
Source: Curr Oncol. 2024 Jan 11;31(1):436–46. doi: 10.3390/curroncol31010029 (PMC10814981; doi:10.3390/curroncol31010029)

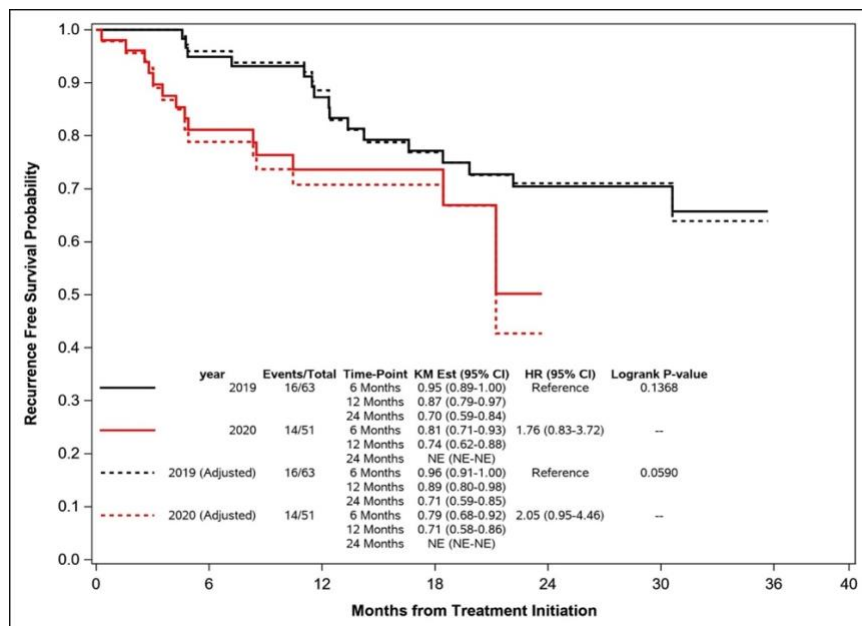

Supplement: Supplementary file 1 [file curroncol-31-00029-s001.zip › curroncol-2769224-supplementary.pdf]
